# Risk prediction for gastrointestinal bleeding in pediatric Henoch-Schönlein purpura using an interpretable transformer model

**DOI:** 10.3389/fphys.2025.1630807

**Published:** 2025-10-02

**Authors:** Gahao Chen, Ziwei Yang

**Affiliations:** The Department of Pediatrics at the Affiliated Hospital of North Sichuan Medical College, NanChong, Sichuan, China

**Keywords:** Henoch-Schönlein purpura, machine learning, transformer architecture, interpretability, gastrointestinal bleeding

## Abstract

**Objective:**

Henoch-Schönlein purpura (HSP), clinically recognized as IgA vasculitis (IgAV), a prevalent systemic vasculitis in pediatric populations, frequently involves gastrointestinal (GI) tract manifestations that may lead to serious complications including hemorrhage and tissue necrosis. Timely identification of GI bleeding risk enables prompt clinical intervention and improves therapeutic outcomes. This study aims to develop and clinically validate an interpretable Transformer-based predictive model for assessing GI bleeding risk in pediatric patients with IgAV.

**Methods:**

This retrospective cohort study analyzed 758 pediatric IgAV cases (ages 0–14 years) admitted to the Department of Pediatrics at the Affiliated Hospital of North Sichuan Medical College between 1 May 2020, and 31 January 2024. Comprehensive clinical data including symptoms and laboratory parameters were systematically collected. GI complications were stratified into three severity tiers: 1) no complications, 2) abdominal pain without bleeding), and 3) documented rectal bleeding or hemorrhage, based on standardized diagnostic criteria. Five machine learning algorithms (Random Forest, XGBoost, LightGBM, CatBoost, and TabPFN-V2) were optimized through nested cross-validation. Model performance was evaluated using multiple metrics: accuracy, precision, recall, *F1*-score, the *Kappa* coefficient, and ROC-AUC. The optimal model was subsequently interpreted using Shapley Additive Explanations (SHAP) values to elucidate feature importance.

**Results:**

Among the evaluated models, the Transformer-based TabPFN-V2 demonstrated superior predictive performance, achieving a validation accuracy of 0.88, precision of 0.88, recall of 0.87, *F1*-score of 0.88, *Kappa* coefficient of 0.82, and AUC-ROC of 0.98. SHAP analysis revealed the five most influential biomarkers for global interpretability: D-dimer, total cholesterol, platelet count, apolipoprotein, and C-reactive protein.

**Conclusion:**

The interpretable Transformer-based TabPFN-V2 model demonstrated robust predictive performance for GI bleeding risk in pediatric IgAV patients. Clinically accessible laboratory parameters identified by this model not only offer practical guidance for clinical decision-making but also establish a foundation for advancing medical artificial intelligence integration in pediatric care.

## Introduction

IgA vasculitis (IgAV), the most prevalent childhood systemic vasculitis, primarily affects small blood vessels in multiple organ systems including the skin, gastrointestinal (GI) tract, joints, and kidneys (Parums, 2024). Among these, GI complications - particularly abdominal pain and bleeding - represent significant clinical challenges (Castañeda et al., 2024; Li et al., 2024). While common in IgAV, severe GI hemorrhage can dramatically elevate pediatric mortality rates and serves as an independent risk factor for subsequent renal impairment (Carucci et al., 2022). The assessment of GI involvement remains clinically problematic due to several factors: 1) the subjective nature of pediatric pain reporting, 2) difficulties in objectively quantifying symptom severity, and 3) the current reliance on extensive laboratory testing for definitive diagnosis. These limitations frequently result in delayed diagnosis and treatment initiation, potentially exacerbating disease progression while simultaneously increasing the financial burden on affected families (Jhaveri et al., 2023). Ultimately, such diagnostic delays may adversely impact long-term patient outcomes.

Currently, predictive models for GI bleeding in IgAV primarily rely on conventional machine learning (ML) approaches, particularly random forest algorithms (Guo et al., 2025). However, these traditional methodologies face significant limitations in processing high-dimensional datasets with numerous features, potentially compromising both predictive accuracy and clinical utility. Furthermore, existing models often fail to adequately capture the intricate interplay among multiple clinical and laboratory parameters, resulting in suboptimal predictive performance (Nie et al., 2023). Consequently, the development and selection of more sophisticated modeling frameworks specifically optimized for tabular medical data represents a critical step toward enhancing both predictive capabilities and clinical decision-making in this domain.

TabPFN represents a transformer-based architecture specifically designed to enhance traditional ML algorithms for tabular data processing (Hollmann et al., 2025). The framework implements a bidirectional hierarchical attention mechanism that enables integrated processing of both categorical and numerical features. The architecture uniquely combines In-Context Learning with Bayesian inference, creating an efficient bridge between Bayesian methodologies and deep learning frameworks. The core innovation of TabPFN lies in its reformulation of posterior approximation as a supervised learning task. TabPFN architecture demonstrates substantial advancements through pre-training on a comprehensive 130-million synthetic tabular prediction dataset while achieving state-of-the-art inference efficiency. As a groundbreaking paradigm in tabular learning, TabPFN has emerged as one of the most significant foundational models in the field. Its 2025-optimized iteration, TabPFN-V2, further advances these capabilities.

The inherent opacity of ML algorithms (often referred to as “black box” characteristics) presents a significant barrier to clinical adoption, as it undermines trust among both patients and healthcare providers (Martin et al., 2023). To address this critical challenge, SHapley Additive exPlanations (SHAP) has emerged as a powerful interpretability framework rooted in game theory principles (Chen, 2025). Within this framework, Kernel SHAP represents a particularly valuable model - agnostic interpretation technique - a specialized variant of Local Interpretable Model-agnostic Explanations (LIME). This approach quantifies feature importance by computing Shapley values, thereby elucidating how each input variable contributes to the model’s predictions. These characteristics make Kernel SHAP particularly valuable in medical contexts, enabling clinicians to both understand prediction mechanisms and optimize early intervention strategies (Feng et al., 2023).

This study seeks to develop and validate a novel clinical prediction model for GI bleeding in patients with IgAV by leveraging state-of-the-art interpretable Transformer architectures. Our research employs advanced ML techniques to enhance both predictive performance and model transparency.

## Methods

### Study population

This retrospective cohort study utilized clinical data from pediatric patients diagnosed with IgAV at the Affiliated Hospital of North Sichuan Medical College in Nanchong, Sichuan Province, China, between 1 May 2020 and 31 January 2024. Ethical approval for this study was waived by the Institutional Review Board of North Sichuan Medical College Affiliated Hospital due to the retrospective nature of the research and the use of anonymized patient data. The research protocol strictly adhered to the TRIPOD + AI reporting guidelines (Collins et al., 2024) for predictive model studies (Supplementary Table S1). Notable limitations include its single-center design and current absence of external population validation, which necessitates cautious interpretation when extrapolating findings to broader demographics. To ensure transparency and reproducibility, the complete source code and optimized model parameters are openly accessible via GitHub (repository: https://github.com/zhuzhuchifei/HSP24). The prediction model has been successfully deployed in our clinical laboratory and is slated for multicenter external validation in Q2 2026, pending ethical approvals.

### Database

According to the European Alliance of Associations for Rheumatology (EULAR) diagnostic criteria for IgAV (Ozen et al., 2019), definitive diagnosis requires the presence of palpable purpura predominantly affecting the lower extremities, accompanied by at least one of the following manifestations: 1) acute diffuse or localized abdominal pain with associated GI symptoms; 2) arthralgia/arthritis confirmed by clinical examination or ultrasonographic evidence of joint inflammation; 3) renal involvement manifested as hematuria and/or proteinuria. Abdominal complications were specifically defined as meeting any of the following criteria: 1) acute abdominal pain requiring medical intervention; 2) melena or hematochezia; 3) hematemesis; 4) persistent vomiting; 5) radiologically confirmed intussusception; 6) occult blood-positive stools. Exclusion criteria comprised: 1) receipt of glucocorticoids or immunosuppressive therapy within 7 days prior to admission; 2) documented history of peptic ulcer disease; 3) evidence of secondary vasculitis; 4) incomplete medical records.

Building upon established evidence in IgAV research literature (Li et al., 2023; Yang et al., 2022; Shen et al., 2025), clinical data were systematically extracted from the hospital’s electronic medical record system, encompassing demographic characteristics, clinical manifestations, laboratory tests, ultrasonography, and gastroscopy. The variance filtering method is employed for feature selection, wherein low-variance features (threshold = 0.1) are removed after data normalization to achieve dimensionality reduction. According to the severity of GI bleeding symptoms, patients were divided into three groups based on 21 variable data including: age (year), gender, white blood cell count (WBC), neutrophil count (GR), lymphocyte count (LY), hemoglobin (Hb), platelets (PLT), aspartate aminotransferase (AST), alanine aminotransferase (ALT), albumin (ALB), total cholesterol (TC), apolipoprotein (APO), lipoprotein a (Lp-a), C-reactive protein (CRP), homocysteine (HCY), lactate (LA), lactate dehydrogenase (LDH), hydroxybutyrate dehydrogenase (LDHD), creatine kinase (CK), creatine kinase-MB isoenzyme (CKMB) and D-dimer (D-D). Patients were categorized into three distinct groups based on GI manifestations: 1) No-GIS group: Complete absence of GI symptoms; 2) Mild-GIS group: Exhibiting transient GI symptoms including intermittent abdominal pain or isolated episodes of emesis; 3) Severe-GIS group: Characterized by significant GI complications including clinically evident GI hemorrhage or radiologically confirmed intestinal edema or documented cases of intussusception. All data were collected from hospital medical records within the 9 days preceding glucocorticoid treatment. After applying these rigorous selection criteria, our final cohort comprised 758 eligible IgAV cases for retrospective analysis.

### Approaching the issue of missing data

Analysis confirms that missing values constitute less than 5% of the total dataset. Imputation methodology was determined by distributional characteristics: variables demonstrating approximate normality (AST, ALB, APO, HCY, LA) underwent mean imputation to preserve parametric properties and central tendency, while non-normally distributed variables (ALT, TC, Lp, CRP, LDH, LDHD, CK, CKMB) necessitated median imputation due to its comparative robustness against outlier-induced bias. For categorical variables, mode imputation was implemented to maintain the original frequency distribution of nominal features. Furthermore, we continuously monitor model fairness using ‌IBM’s AI Fairness 360 (AIF360) toolkit (Bellamy et al., 2019), systematically identifying and mitigating biases - particularly those related to gender and age disparities - during model analysis.

### Statistical analyses

The Kruskal–Wallis *H* test serves as a non-parametric method for comparing laboratory parameters across multiple independent groups, with statistically significant results prompting subsequent pairwise comparisons. For algorithm performance evaluation, the Friedman test is implemented through Orange3 library (version 3.32.0) to detect potential significant differences among multiple algorithms. Upon identifying statistically significant variations, the Nemenyi *post hoc* test is employed to precisely quantify performance disparities between any two given algorithms. This follow-up test operates by calculating the critical range for differences in average rank values, whereby a measured difference exceeding this threshold indicates statistically significant performance divergence. All statistical interpretations adhere to the conventional significance threshold of *P* < 0.05.

### Development and validation of predictive models

**FIGURE 1:**
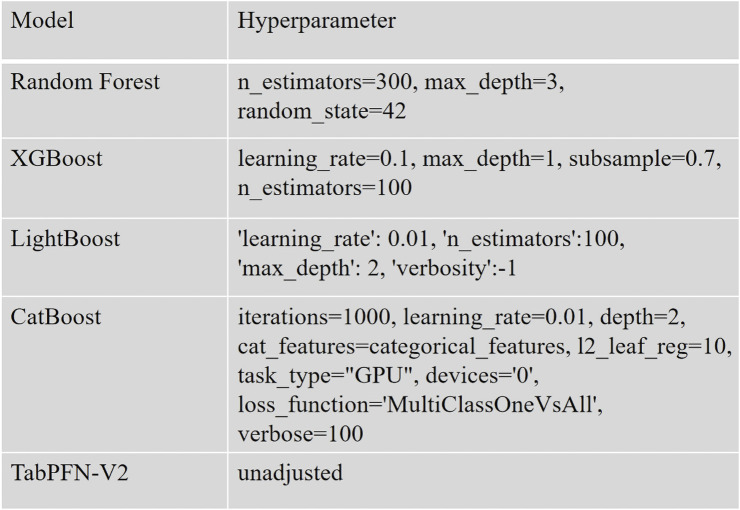
Selection of hyperparameters for the model.

The data preprocessing and analysis were performed using Python 3.10 along with the scikit-learn library (version 1.4.2). For ML modeling, we implemented several supervised algorithms including Random Forest (from scikit-learn), XGBoost (version 1.7.3), LightGBM (version 4.1.0), CatBoost (version 1.2), and TabPFN (version 2.0). To comprehensively evaluate model performance, we employed multiple metrics: accuracy, precision, recall, *F1-*score, and the *Kappa* coefficient. A nested cross-validation (Outer layer k = 5, Inner layer k = 2) strategy was adopted to mitigate overfitting risks and enhance model robustness, ultimately enabling the selection of the optimal performing model (Figure 1).

Given the categorical nature of the target variable, model performance was assessed using both classification accuracy and Cohen’s *Kappa* coefficient. The *Kappa* coefficient serves as a robust metric for evaluating classification consistency beyond chance agreement. Its calculation involves two critical components: Observed Agreement (*P*_*0*_): The proportion of correctly classified instances, calculated as the sum of diagonal elements in the confusion matrix divided by the total number of samples. Expected Agreement (*P*_*e*_): The hypothetical probability of random agreement, computed by summing the product of corresponding row and column marginal probabilities across all categories. This normalization approach ensures *Kappa* values range between −1 and 1, with higher values indicating stronger model performance independent of class distribution in Table 1.

**TABLE 1:** The meaning of the *Kappa coefficient*.

*Kappa coefficient*	−1	0	0.0∼0.20	0.21∼0.40	0.41∼0.60	0.61∼0.80	0.81∼1
Meaning	Complete inconsistency	Accidental consistency	Extremely low consistency	General consistency	Moderate consistency	High consistency	Almost identical

The final dataset was partitioned into training and validation subsets using an 80:20 ratio, with random_state = 42 initialized to ensure experiment reproducibility.

### Tools for interpreting machine learning

We employ SHAP version 0.42.1 to interpret the optimal prediction model. The Kernel SHAP framework is implemented to conduct both global and local model interpretations. Feature importance ranking is determined by computing SHAP values, with features ordered according to their mean absolute SHAP values. This integration of ML with SHAP explanation methods establishes a robust theoretical foundation for predictive modeling by providing transparent, quantitative insights into model behavior.

## Results

### Clinical characteristics

This retrospective study enrolled 758 pediatric patients diagnosed with IgAV, with a mean age of 7.2 ± 2.5 years. The cohort comprised 441 males (58.2%) and was stratified into three clinical subgroups: 303 cases (40.0%) without GI manifestations, 231 cases (30.5%) presenting with abdominal pain, and 224 cases (29.6%) exhibiting rectal bleeding. The time interval from IgAV symptom onset to hospital admission for laboratory evaluation ranged from 0 to 7 days (mean: 2.7 days). Subsequent progression to GI bleeding occurred within 1–9 days post-admission (mean: 3.5 days). As detailed in Table 2, statistically significant intergroup differences (*P* < 0.05) were observed across multiple laboratory parameters, including hematological markers (WBC, GR, Hb, PLT), biochemical function markers (AST, ALB, LDH, LDHD), lipid profile (TC, APO), and inflammatory/coagulation markers (CRP, D-D).

**TABLE 2:** Demographics of research population.

Variable	Non GIB(n = 303)	Mild GIB(n = 231)	Severe GIB(n = 224)	*P value*
Age (y)	7.14 ± 2.53	7.12 ± 2.49	7.22 ± 2.42	0.835
Male	180	133	128	0.852^a^
WBC (×10^9^/L)	9.94 ± 3.38	11.08 ± 4.54	12.99 ± 5.38	**0.000**
GR (×10^9^/L)	6.73 ± 3.06	7.61 ± 3.99	8.81 ± 4.65	**0.000**
LY (×10^9^/L)	2.60 ± 1.51	2.79 ± 1.69	2.68 ± 1.68	0.669
Hb(g/L)	123.76 ± 10.06	126.30 ± 12.89	129.53 ± 13.70	**0.000**
PLT (×10^9^/L)	296.80 ± 81.76	350.04 ± 110.37	354.01 ± 104.69	**0.000**
AST (U/L)	29.94 ± 8.47	31.98 ± 12.21	28.66 ± 9.35	**0.047**
ALT (U/L)	15.21 ± 15.81	15.24 ± 12.05	13.79 ± 6.25	0.209
ALB (g/L)	41.51 ± 5.15	40.26 ± 5.36	41.22 ± 7.35	**0.013**
TC (mmol/L)	3.76 ± 0.75	4.36 ± 2.48	4.04 ± 0.79	**0.004**
APO (g/L)	0.62 ± 0.17	0.72 ± 0.19	0.75 ± 0.22	**0.000**
Lp (mg/L))	212.18 ± 196.88	212.39 ± 191.89	281.13 ± 254.32	0.055
CRP (mg/L)	9.68 ± 12.04	7.04 ± 5.97	10.68 ± 9.43	**0.001**
HCY(mmol/L)	7.68 ± 2.49	7.68 ± 3.23	7.62 ± 3.52	0.804
LA (mmol/L)	2.74 ± 1.01	2.73 ± 1.09	2.69 ± 1.08	0.831
LDH (U/L)	212.00 ± 48.95	214.18 ± 76.74	194.89 ± 59.21	**0.000**
LDHD (U/L)	172.76 ± 44.05	196.79 ± 89.17	157.95 ± 65.93	**0.000**
CK (U/L)	71.42 ± 59.69	95.64 ± 114.84	60.03 ± 32.99	0.423
CKMB (U/L)	12.56 ± 7.49	14.09 ± 9.85	13.49 ± 9.49	0.419
D-D (mg/L)	1.04 ± 0.59	1.51 ± 0.41	2.75 ± 1.05	**0.000**

GIB, gastrointestinal bleeding; WBC, white blood cell; GR, neutrophil count; LY, lymphocyte count; Hb, Hemoglobin; PLT, platelet count; AST, aspartate aminotransferase; ALT, alanine aminotransferase; ALB, albumin; TC, total cholesterol; APO, apolipoprotein; Lp-a; Lipoprotein-a; CRP, C-reactive protein; HCY, homocysteine; LA, lactate; LDH, lactate dehydrogenase; LDHD, lactate dehydrogenase D; CK, creatine kinase; CK-MB, Creatine kinase-MB, isoenzyme; D-D, D-dimer. Boldface indicates a statistically significant difference with P < 0.05. a Chi-square test. The bolded values highlight statistically significant P-values.

Post-hoc pairwise comparisons were performed for all parameters demonstrating statistical significance. Table 3 further demonstrates statistically significant differences in the inter-group comparisons.

**TABLE 3:** Post-hoc pairwise comparisons.

Variable	Non GIB vs. Mild GIB	Non GIB vs. ‌Severe GIB	Mild GIB vs. ‌Severe GIB
WBC	**0.014**	**0.000**	**0.000**
GR	**0.041**	**0.000**	**0.009**
Hb	0.091	**0.000**	**0.026**
PLT	**0.000**	**0.000**	1.000
AST	0.602	**0.041**	0.521
ALB	1.000	**0.017**	0.103
TC	0.735	**0.003**	0.133
APO	**0.000**	**0.000**	0.413
CRP	0.613	**0.000**	**0.017**
LDH	1.000	**0.000**	**0.000**
LDHD	**0.003**	**0.000**	0.113
D-D	**0.000**	**0.000**	**0.000**

The bolded values highlight statistically significant P-values.

Given the variations in normal reference ranges for hematological markers across different age groups, we performed comparative analyses of WBC, GR, HB, and PLT among these demographic cohorts (Table 4).

**TABLE 4:** Comparison of grouping hematological markers for different age groups.

Variable	0–3 years old (n = 54)	4–6 years old (n = 268)	7–9 years old (n = 294)	10–14 years old (n = 142)	*P value*
WBC	10.95 ± 0.52	10.75 ± 0.26	11.42 ± 0.27	11.39 ± 0.43	0.302
GR	7.50 ± 0.41	7.45 ± 0.23	7.78 ± 0.23	7.65 ± 0.39	0.623
LY	2.67 ± 0.22	2.52 ± 0.09	2.74 ± 0.09	2.92 ± 0.14	0.120
Hb	128.02 ± 1.65	126.59 ± 0.70	125.48 ± 0.75	126.46 ± 1.09	0.257
PCT	343.81 ± 12.44	328.04 ± 6.13	327.82 ± 6.11	332.59 ± 8.55	0.724

### Machine learning model performance

Through comprehensive evaluation of 5 ML models across both training and validation sets, TabPFN-V2 demonstrated superior performance metrics. The model achieved consistently high scores in the validation set, with accuracy 0.88, sensitivity 0.88, recall 0.88, *F1-*score 0.87, and the *Kappa* coefficient 0.82, outperforming all other compared models. (Table 5).

**TABLE 5:** Machine learning model performance.

Model	Training				Validation				
	Accuracy	Precision	Recall	*F1*-score	Accuracy	Precision	Recall	*F1*-score	*Kappa*
RF	0.79	0.86	0.77	0.79	0.78	0.82	0.77	0.78	0.66
XGBoost	0.86	0.88	0.84	0.85	0.80	0.83	0.81	0.80	0.70
LightGBM	0.77	0.86	0.73	0.75	0.70	0.83	0.73	0.72	0.69
CatBoost	0.82	0.85	0.79	0.81	0.77	0.82	0.79	0.77	0.66
TabPFN	**0.95**	**0.96**	**0.95**	**0.95**	**0.88**	**0.88**	**0.88**	**0.87**	**0.82**

The bolded values reflect the most favorable performance outcomes.

**FIGURE 2:**
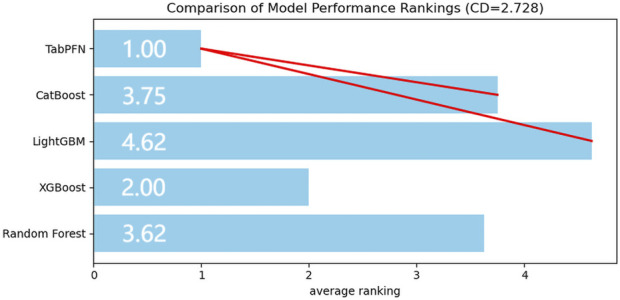
Average ranking of model performance.

The accuracy, sensitivity, recall, and F1-score of both training and validation sets from five machine learning models were evaluated across eight performance dimensions using the Friedman test. The analysis yielded statistically significant results (χ^2^ = 26.624, *P* < 0.01). Subsequent Nemenyi *post hoc* testing revealed that the TabPFN-V2 model achieved the highest mean rank, with statistically significant differences (*P* < 0.05) observed between TabPFN, CatBoost, and LightGBM (Figure 2).

### Area under the multi-class ROC curve

**FIGURE 3:**
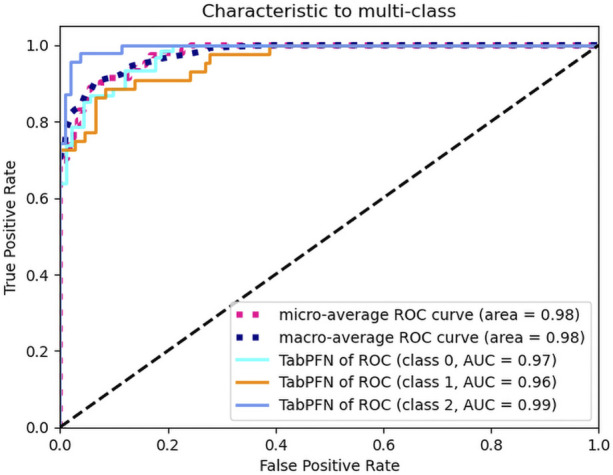
Area under the multi-class ROC curve.

The TabPFN model demonstrated exceptional discriminatory performance, with both macro-average and micro-average ROC-AUC scores reaching 0.98. When evaluated using a OvR strategy across clinical subgroups, the model maintained consistently high AUC values: 0.97 for asymptomatic cases, 0.96 for the abdominal pain subgroup, and 0.99 for the bleeding subgroup. These outstanding metrics strongly suggest that our selected features serve as robust predictors for GI complications in pediatric IgAV cases. The TabPFN model therefore represents an optimal choice for classification modeling with this dataset (Figure 3).

### The transformer model interpretation with kernel SHAP methods

**FIGURE 4:**
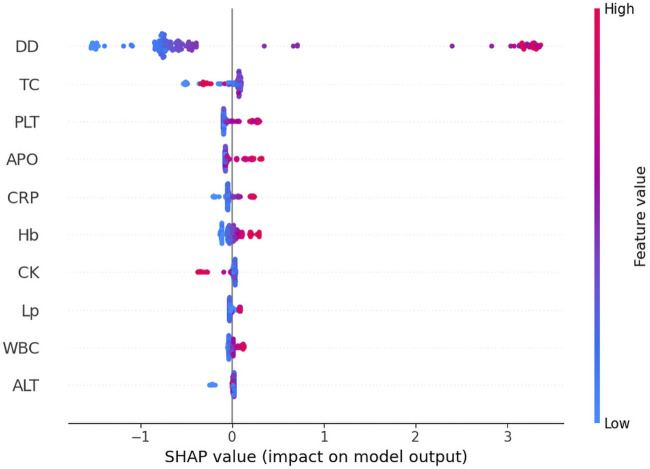
The global interpretability based on Kernel SHAP under the transformer model. D-D, D-dimer; TC, Total cholesterol; PLT, Platelet count; APO, Apolipoprotein; CRP, C-reactive protein; Hb, Hemoglobin; CK, Creatine kinase; Lp-a; Lipoprotein-a; WBC, White blood cell; ALT, Alanine aminotransferase; SHAP, SHapley Additive exPlanations.

The global interpretability analysis using Kernel SHAP reveals the feature importance ranking, highlighting the top five most influential variables. Each feature is represented by a distinct horizontal line, with colored data points (red indicating high contribution values and blue denoting low values) showing individual patient results (Figure 4). The analysis identifies the following key predictors for IgAV GI bleeding risk: D-dimer level, total cholesterol, platelet count, apolipoprotein, and C-reactive protein level.

**FIGURE 5:**
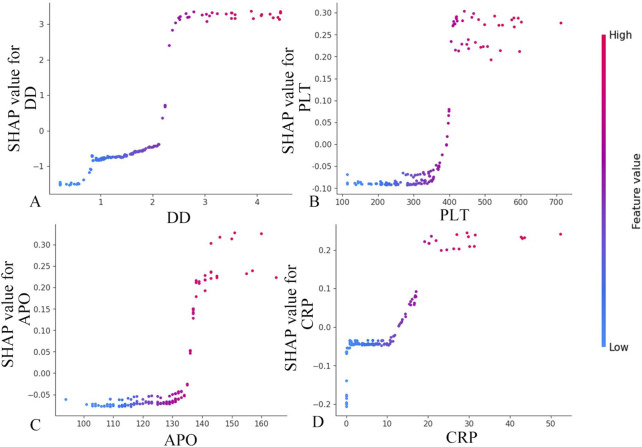
Feature dependence plots. **(A)** Feature dependence plots of D-dimer. **(B)** Feature dependence plots of platelet count. **(C)** Feature dependence plots of apolipoprotein. **(D)** Feature dependence plots of CRP. The horizontal axis quantifies the parameter’s numerical range, while the vertical axis corresponds to the computed Shapley values. The color gradient reflects parameter importance, with increasing red saturation indicating higher marginal contributions of specific parameter values to the model’s predictive performance.

Figure 5 demonstrates a positive correlation between biomarker levels (D-dimer, platelet count, apolipoprotein, and CRP) and GI symptoms, as evidenced by progressively elevated SHAP scores with increasing parameter values. The observed S-shaped transition curves suggest a critical threshold phenomenon, where surpassing specific biomarker thresholds triggers disease progression to hemorrhagic stages. These nonlinear relationships provide valuable insights for developing a risk stratification system, informing both diagnostic thresholds and prognostic evaluation in IgAV related GI complications.

**FIGURE 6:**
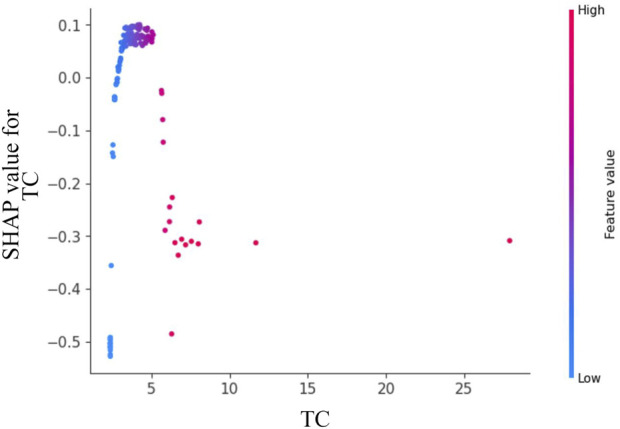
Feature dependence plots of total cholesterol. The horizontal axis quantifies the parameter’s numerical range, while the vertical axis corresponds to the computed Shapley values. The color gradient reflects parameter importance, with increasing red saturation indicating higher marginal contributions of specific parameter values to the model’s predictive performance.

Figure 6 reveals a dose-dependent relationship between TC levels and Shapley values, where increasing TC concentrations correlate with progressively higher SHAP scores. The predictive contribution peaks at a critical TC threshold of approximately 5 mmol/L, beyond which the SHAP values demonstrate a gradual attenuation pattern. This biphasic response suggests a potential saturation effect in TC’s pathological contribution to disease progression.

## Discussion

This study develops a transformer based prediction model for GI complications in pediatric IgAV patients, comparing five algorithmic approaches. Our results demonstrate that the TabPFN model outperforms conventional methods (Random Forest, LightGBM, XGBoost, and CatBoost) in distinguishing between IgAV related abdominal pain and GI, exhibiting superior predictive capabilities. Current predictive methodologies for IgAV related GI bleeding predominantly rely on univariate analysis (*P* < 0.05 threshold) followed by multivariate logistic regression modeling (Su et al., 2025; Yang et al., 2024). While logistic regression remains a fundamental linear approach, its limitations in handling nonlinear relationships often lead to exclusion of potentially valuable variables that fail to meet traditional statistical significance criteria. Recent literature has highlighted ML’s potential in predicting IgAV outcomes, which our findings substantiate (Guo et al., 2025).

Within our cohort of IgAV patients, 224 cases (29%) presented with GI, aligning with established epidemiological data (Sağlam et al., 2025). The TabPFN model achieved exceptional performance metrics (accuracy, precision, recall, *F1*-score, and the *Kappa* coefficient) during internal validation, with a macro-average ROC of 0.98. Further demonstrating the superiority of the TabPFN model through rigorous statistical validation using Friedman’s test and Nemenyi’s test. Systematically monitor model fairness using AIF360 to ensure ethical compliance with global standards and mitigate bias arising from data scarcity. This robust predictive capability enables early identification of high-risk patients, allowing clinicians to implement timely interventions during critical treatment windows to mitigate bleeding risks. While promising, the model requires external validation to confirm its generalizability across diverse clinical settings. We have successfully implemented a parameterized model in the local clinical laboratory. Future work will focus on integrating the model into the clinical decision support system or deploying it via Streamlit to facilitate multicenter external validation.

The development of TabPFN stems from the inherent limitations of conventional ML approaches in handling tabular data, particularly when dealing with dataset heterogeneity and raw data complexity. The 2025 release of TabPFN-v2 introduces significant functional enhancements, expanding its capabilities to include not only improved categorical variable analysis but also inaugural support for regression tasks. Notably, the model natively accommodates missing values and outliers without requiring manual feature engineering. With optimal performance on small-to-medium datasets (≤10,000 samples and ≤500 features), TabPFN-v2 demonstrates superior accuracy compared to existing methods while achieving substantially reduced training times. Built on a Generative Transformer architecture, this foundational model supports multiple advanced functionalities including fine-tuning, synthetic data generation, density estimation, and learnable embedding extraction. TabPFN’s unique training paradigm leverages millions of synthetic datasets, showcasing remarkable algorithmic development capabilities. By advancing modeling proficiency across diverse domains, this innovation holds significant potential to accelerate scientific breakthroughs and enhance decision-making processes in various fields.

The clinical application of ML models is frequently constrained by their inherent lack of interpretability, raising concerns about their reliability in predicting disease outcomes (Hofweber and Walker, 2024). To address this challenge, we employed SHAP analysis on the TabPFN-V2 model, utilizing Kernel SHAP methodology to interpret and visualize prediction results. Our analysis identified five significant biomarkers for IgAV associated GI bleeding: D-dimer, total cholesterol, platelet count, apolipoprotein, and C-reactive protein.

The rising incidence of pediatric IgAV with GI complications has heightened the need for early risk identification. This condition presents diagnostic challenges due to nonspecific early symptoms, frequent misdiagnosis, and high recurrence rates (Kato et al., 2024). In this study, we demonstrated that elevated D-dimer levels (>2 mg/L) and elevated platelet counts (>400 × 10^9^/L) exhibited significantly enhanced marginal contributions within the predictive model. Previous studies have indicated that the pathogenesis of gastrointestinal bleeding in children with IgAV involves the synergistic effect of increased platelet aggregation and D-dimer-induced microthrombus formation (Su et al., 2025). This threshold effect analysis quantitatively confirms that surpassing these biomarker thresholds substantially elevates the risk of GI bleeding in pediatric IgAV patients. The pathogenesis of GI hemorrhage appears multifactorial (Wei et al., 2023), with our findings specifically establishing that concurrent APO exceeding 130 g/L and CRP levels exceeding 20 mg/L collectively elevate pediatric GI bleeding risk. A study found that serum APO levels are elevated in patients with lgAV (Wu et al., 2019). However, in patients with IgAV Nephritis (IgAVN), apoM loss due to kidney injury results in decreased serum APO concentrations. Furthermore, these apoM levels decline progressively with worsening renal impairment and show a significant inverse correlation with ISKDC (the International Study of Kidney Disease in Children) grading scores in IgAVN patients. Current evidence remains inconclusive regarding the specific role of APO in the pathogenesis of IgAV GI bleeding, warranting further investigation to elucidate potential mechanistic links. Through SHAP analysis, it was conclusively demonstrated that D-dimer, platelet count, APO, and C-reactive protein serve as pivotal biomarkers for IgAV GI prediction. By establishing optimized diagnostic thresholds for these parameters, clinicians can significantly enhance model predictive performance. Notably, this approach provides pediatricians with quantifiable decision support, facilitating more accurate and objective assessment of pediatric IgA vasculitis cases.

Chen et al. (2024) conducted a systematic investigation into the relationship between the Dietary Inflammation Index (DII) and IgAV, demonstrating that dietary factors exert measurable influence on disease severity and complication development in pediatric IgAV patients. Their study revealed statistically significant associations between higher DII scores and multiple clinical indicators, including elevated inflammatory biomarkers, suboptimal nutrient intake profiles, dysregulated lipid metabolism parameters, and increased complication rates. These findings not only systematically delineate the mechanistic pathways connecting pro-inflammatory dietary patterns with IgAV pathophysiology at the molecular level, but also rigorously establish an evidence-based framework for developing precision nutrition models.

Notably, blood TC levels emerged as the most straightforward predictive indicator for IgAV related abdominal pain and GI bleeding. Emerging evidence has established a compelling association between dyslipidemia and the clinical trajectory of immunoglobulin A nephropathy (IgAN), with particular emphasis on hypertriglyceridemia and hypercholesterolemia exacerbating hypertension and proteinuria - pivotal determinants of disease progression (Nüsken and Weber, 2022). Wang’s seminal work demonstrated a markedly diminished renal survival rate in IgAN patients with concomitant hypertriglyceridemia, underscoring its prognostic significance as an independent risk factor (Wang et al., 2020). While lipid research has predominantly centered on IgAN populations, IgAV remains comparatively understudied. Importantly, the lipid spectrum encompasses diverse constituents including fats, phospholipids, and steroids. Our SHAP-based analysis identified TC as a model feature with substantial marginal contribution, exhibiting a dose-response relationship where SHAP scores peak at approximately 5 mmol/L before plateauing. This observation suggests that early dyslipidemia in IgAV may serve as a predictive biomarker for GI complications, thereby facilitating timely endoscopic assessment and therapeutic intervention. Through optimal threshold selection for TC, we enhanced the performance of our single-variable prediction model. The kernel SHAP analysis provides clinicians with two key advantages: 1) comprehensive risk factor visualization that supplements standard model outputs, and 2) personalized explanatory insights into the model’s decision-making process. While these interpretability techniques represent significant progress, we acknowledge SHAP’s methodological limitations and emphasize the need for further validation of kernel SHAP approaches in clinical practice. Meanwhile, the current findings should be interpreted cautiously given the study’s single-center design, modest sample size, and inherent limitations of retrospective analyses.

This study acknowledges several noteworthy limitations that warrant careful consideration. First, while our cohort size exceeds those reported in prior studies, it remains suboptimal relative to the data requirements of modern ML algorithms, potentially limiting model generalizability. Second, Given the inherent variability in dietary patterns and genetic predispositions across populations, the generalizability of these study findings may be constrained. Therefore, future investigations should prioritize multicenter studies involving diverse ethnic cohorts to validate these observations, thereby strengthening the translational applicability of the research outcomes. This approach will facilitate the development of more universally relevant clinical guidelines and intervention strategies. Third, despite TabPFN-V2’s established versatility in tabular data processing, the single-institution provenance of our dataset raises concerns regarding clinical translatability, necessitating rigorous external validation across multi-center cohorts with geographic and demographic diversity. A critical limitation of SHAP lies in its inherent inability to distinguish correlation from causation. While SHAP analysis enhances model interpretability, the computational complexity of kernel SHAP based logical operation interpretation presents substantial challenges in clinical deployment contexts, demanding prohibitive temporal and hardware resources. This underscores the need to investigate emerging interpretability frameworks specifically optimized for medical applications, such as the SHAP-IQ, which may offer pediatricians more clinically actionable insights through enhanced visualization capabilities.

## Conclusion

Our transformer based algorithm integrates multidisciplinary technologies with medical database information to establish an early warning system for IgAV associated GI bleeding in pediatric patients, enabling personalized treatment and preventive care. The study revealed multiple laboratory markers significantly correlated with IgAV related GI complications, advancing our understanding of IgAV pathophysiology and facilitating predictive model development for clinical guidance. The transformer architecture TabPFN-V2 model demonstrated exceptional performance in this application. Routine laboratory tests, serving as readily accessible parameters, offer valuable clinical references, empowering pediatricians to effectively identify high-risk IgAV patients and optimize GI bleeding management. This approach demonstrates substantial clinical significance and lays a practical foundation for advancing medical-artificial intelligence integration.

## Data Availability

The datasets presented in this study can be found in online repositories. The names of the repository/repositories and accession number(s) can be found below: https://github.com/zhuzhuchifei/HSP24.
